# CacyBP/SIP promotes the proliferation of colon cancer cells

**DOI:** 10.1371/journal.pone.0169959

**Published:** 2017-02-14

**Authors:** Huihong Zhai, Yongquan Shi, Xiong Chen, Jun Wang, Yuanyuan Lu, Faming Zhang, Zhengxiong Liu, Ting Lei, Daiming Fan

**Affiliations:** State Key Laboratory of Cancer Biology, Institute of Digestive Diseases, Xijing Hospital, Fourth Military Medical University, Xi’an, China; Sun Yat-sen University Zhongshan School of Medicine, CHINA

## Abstract

CacyBP/SIP is a component of the ubiquitin pathway and is overexpressed in several transformed tumor tissues, including colon cancer, which is one of the most common cancers worldwide. It is unknown whether CacyBP/SIP promotes the proliferation of colon cancer cells. This study examined the expression level, subcellular localization, and binding activity of CacyBP/SIP in human colon cancer cells in the presence and absence of the hormone gastrin. We found that CacyBP/SIP was expressed in a high percentage of colon cancer cells, but not in normal colonic surface epithelium. CacyBP/SIP promoted the cell proliferation of colon cancer cells under both basal and gastrin stimulated conditions as shown by knockdown studies. Gastrin stimulation triggered the translocation of CacyBP/SIP to the nucleus, and enhanced interaction between CacyBP/SIP and SKP1, a key component of ubiquitination pathway which further mediated the proteasome-dependent degradation of p27^kip1^ protein. The gastrin induced reduction in p27^kip1^ was prevented when cells were treated with the proteasome inhibitor MG132. These results suggest that CacyBP/SIP may be promoting growth of colon cancer cells by enhancing ubiquitin-mediated degradation of p27^kip1^.

## Introduction

Colon cancer is one of the most common cancers in the world, and one of the leading causes of death. The etiology of colon cancer is multifactorial and has remained poorly defined [1, 2].

Calcyclin-binding protein (CacyBP) interacts with S100A6 (calcyclin) at a physiological range of Ca^2+^ concentrations in Ehrlich ascites tumor cells [3]. Further investigation demonstrated that the Siah-1 Interacting Protein (SIP) is an ortholog of human CacyBP [4], leading SIP to be renamed as CacyBP/SIP. CacyBP/SIP plays important roles in cellular processes such as ubiquitination, proliferation, differentiation, tumorigenesis, cytoskeletal rearrangement and regulation of transcription [5–8]. Two research groups reported that CacyBP/SIP was translocated to the nucleus and phosphorylated when intracellular Ca^2+^ levels are elevated by KCl treatment in neurons and neuroblastoma NB-2a/SH-SY5Y cells [9, 10]. CacyBP/SIP is also a component of the ubiquitin pathway by associating with the Skp1-Cullin1-F-box (SCF) complex and regulating its function [4].

To further understand the function of this protein, our group produced three monoclonal antibodies against CacyBP/SIP [11]. Using these CacyBP/SIP MAbs, we found that CacyBP/SIP was minimally expressed in many normal tissues including colon, but overexpressed in many types of cancerous tissues [12]. In particular, colon cancer cells showed high levels of CacyBP/SIP expression [13]. Further work showed that CacyBP/SIP was translocated to the nucleus by a series of treatment, including KCl, epidermal growth factor, prostaglandin E2 and hypoxia [14–17]. Particularly, gastrin stimulates the nuclear translocation of CacyBP/SIP, which was also correlated with the elevation of [Ca^2+^]*i* and stimulated proliferation of gastric cancer [18]. However, it is not clear whether the nuclear translocation of CacyBP/SIP plays a role in the cell proliferation.

The aim of the present study was to determine whether CacyBP/SIP nuclear translocation is involved in the proliferation of colon cancer cells. We cultured human colon cancer cells, stimulated their proliferation with the carcinogen gastrin and examined the intracellular distribution of CacyBP/SIP. We also tested whether knockdown of CacyBP expression with siRNA affects the ability of gastrin to stimulate proliferation of cancer cells.

## Materials and methods

### Tissue samples

Tissues including tissues from 33 colon adenocarcinoma, 26 noncancerous colons, and 10 normal colons were obtained from the Department of Pathology of General Hospital of Ningxia Medical University, Ningxia, China, fixed with formalin and paraffin-embedded. Four samples of fresh tissue including colon adenocarcinoma and adjacent noncancerous colon tissue were obtained from four surgical patients to perform Western Blot. The diagnoses of the paraffin-embedded and fresh tissue specimens were confirmed by two experienced pathologists. The study was approved by the Ethical Committee of Ningxia Medical University General Hospital.

### Immunohistochemistry

Expression of CacyBP/SIP was examined by immunohistochemistry using a CacyBP/SIP-specific monoclonal antibody prepared by our laboratory [11], at a dilution of 1:150 (clone EA1) with the Envision+ System following the peroxidase method (DAKO, Carpinteria, CA). Pre-immune mouse serum was used instead of primary antibody for negative control. Cells were considered as positive expression if they showed cytoplasmic and/or nuclear staining.

### Cell culture, reagents, and treatment of cells

The human cancer cell lines HT29 (lot no. TCHu103), SW480 (lot no. TCHu172) and Lovo (lot no. TCHu 82) cells were obtained from China cell resource center of academy of life sciences (Shanghai), and maintained in RPMI 1640 (HyClone, Logan, UT) supplemented with 10% FBS (Sijiqing Corp., China). Stably transfected CacyBP/SIPsi cells were cultured in RPMI 1640 medium with 10% FBS and 200 μg/mL G418 (Invitrogen, Carlsbad, CA). Gastrin (Sigma, St. Louis, MO; dissolved in RPMI 1640) was used to treat cells at different final concentrations of 10^−6^, 10^−7^, 10^−8^, 10^−9^ and 10^−10^ mol/L for 24 and 48 hours (h).

### Immunofluorescence analysis

Cells were grown on poly-L-lysine-coated coverslips, fixed with 4% paraformaldehyde, and permeabilized with 0.25% Triton X-100. Cells were then blocked with 1% bovine serum albumin, and probed with anti-CacyBP MAb (1:10) for overnight at 4°C. After washing, cells were incubated with goat anti-mouse IgG-FITC (1:50; Santa Cruz Biotechnology, Santa Cruz, USA), mounted on glass slides,and imaged by a confocal laser microscope (Bio-Rad Laboratories, Inc., USA). As a negative control, cells were incubated with preimmune serum.

### MTT cell proliferation assay

To study the effect of gastrin on CacyBP/SIP in cells, 1×10^4^ cells/well was cultured in 96-well plates for 24 h and subsequently serum-starved for 24 h. Next, gastrin was added to the serum-free medium. After 24 or 48 hrs, cell numbers were quantified using the MTT assay. Cell growth was normalized by the growth of control cell line, which was defined as 100%.

To examine the effect of CacyBP/SIP knockdown on the cell proliferation, 5×10^3^ cells were seeded into a 96-well plate. The plates were incubated for 1d, 3d, 5d, 7d and 9d and MTT test was performed. The proliferation rate of siRNA expressing cells was normalized by the proliferation rate of pSilencer negative control cells, which was always set as 100%.

### Colony formation assays

Cells were seeded with agar in 6-well plates at a density of 200 cells/well and grown in complete medium with or without 10^−8^ mol/L gastrin. After 14 days, plates were washed in PBS, and colonies were fixed with methanol and stained with methylene blue (0.04%). The number of foci >100 μm was counted.

### Cell cycle analysis

50,000 cells per well were seeded in 6-well plates and allowed to grow for 24 h to reach 60–70% confluence. Then, the cells were serum-starved for 24 h and subsequently treated with or without 10^−8^ mol/L gastrin in complete RPMI 1640 medium. 48 h later, cells were fixed overnight in 70% ethanol at 4°C and then incubated in a buffer containing propidium iodide (PI, 50 μg/ml) for 30 min. Cells were analyzed by flow cytometry (EPICS XL, Coulter) with Cell Quest software.

### Western blot analysis

Cells were lysed in 300 μL of freshly prepared extraction buffer [1% SDS, 1 mmol/L Na_3_VO_4_, 150mM NaCl, 0.1 mol/L Tris (pH 7.4)]. Cell fractions were prepared using NE-PER^™^ nuclear and cytoplasmic extraction kit (Pierce Biotechnology Inc., Rockford, IL). Proteins were resolved at 40 μg/lane on 12% SDS-polyacrylamide gels and electrophoretically transferred to polyvinylidene difluoride membranes (Millipore, Bedford, MA). Membranes were blocked and probed with primary antibodies at 4°C overnight. The following monoclonal antibodies were used: p27^kip1^ (1:200) from NeoMarkers (USA); and β-actin (MAb, 1:2,000) from Sigma (St. Louis, MO). The following polyclonal antibodies were used: α-tubulin (1:1,000) and PARP (1:1,000) from Cell Signaling Technology (Boston); and Cyclin E (1:100) from NeoMarkers (USA). Membranes were then incubated with HRP-conjugated goat anti-mouse IgG or HRP-conjugated goat anti-rabbit IgG (Amersham Biosciences, Piscataway, NJ) and detected by Super Signal West Pico Chemiluminescent Substrate (Pierce Biotechnology Inc., Rockford, IL). All of western blots are the representative of three independent sets of immunoblots with similar findings.

### Immunoprecipitation assays

Nuclei extract were prepared using NE-PER^™^ nuclear and cytoplasmic extraction kit. 300 μg of nuclear extract were used for immunoprecipitation by incubation with 1 μg of anti-CacyBP/SIP or anti-GFP (Sigma) and 40 μL of 25% protein A/G agarose slurry (Pierce Biotechnology Inc., Rockford, IL) at 4°C for 2 hours. The protein A/G agarose was recovered by centrifugation at 5,000 rpm and washed four times with ice-cold lysis buffer. Proteins were eluted by mixing with 20 μL of SDS loading buffer and boiling for 5 minutes, followed by immunoblot analysis.

### Construction and transfection of CacyBP/SIP expression vector

CacyBP/SIP was subcloned into the pEGFP-C1 plasmid (Invitrogen, Carlsbad, CA) from the previously constructed plasmid pET28-CacyBP/SIP [11]. The cDNA encoding a mutant of CacyBP/SIP (1–72), containing only the the N-terminal 1–72 residues, was generated by PCR amplification and subcloned into the pEGFP-C1.

Short hairpin RNA (shRNA) specifically targeting CacyBP/SIP was generated using the pSilencer siRNA Construction Kit (Ambion, Austin, TX). The target sequences for CacyBP/SIP were 5’-aagagttactccatgattgtg-3’ (siRNA-1) and 5’- aatcaagaacaagatgcaac-3’ (siRNA-2). We used the pSilencer negative control vector, containing a target sequence that is not found in mouse, human, or rat genome databases.

Cell transfection was performed with Lipofectamine 2000 (Invitrogen, Carlsbad, CA) as described in the manufacturer’s protocol. For transient transfection, cells were harvested at 48 h after transfection for further experiments. For stable transfection, G418 (200 μg/ml) was added to the cells after 24 h of transfection to select the mixed clones.

### Statistical analysis

Numerical data are presented as the mean ± SEM. Differences between means were analyzed with ANOVA and then a post-hoc test. All statistical analyses were performed using SPSS 11.0 software (Chicago, IL, USA). The threshold *p*<0.05 was used to define statistical significance.

## Results

### CacyBP/SIP is overexpressed in colon adenocarcinoma tissues

**Fig 1:**
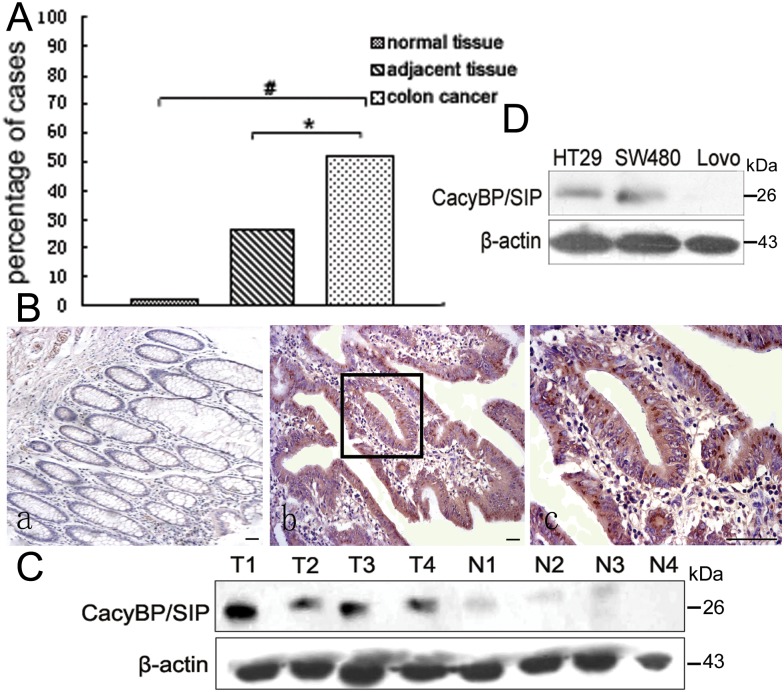
CacyBP/SIP expression in colon cancer and normal colon tissue. (A), percentage of samples showing positive CacyBP/SIP immunoreactivity in normal colon samples, colon cancer tissue, and adjacent noncancerous colon tissue. Cells staining positive were defined as those showing cytoplasmic and/or nuclear staining. (B), representative photomicrographs of CacyBP/SIP staining in normal colon tissue (a, 100×), colon cancer tissue (b, 100×), and a close-up view of panel b showing diffuse cytoplasmic/nuclear staining (c, 400×). Bar = 50 μm. (C) and (D), Western blot analysis of CacyBP/SIP expression in colon cancer tissue and adjacent noncancerous tissue from four surgery patients, as well as the colon cancer cell lines HT29, SW480, and Lovo. All four colon cancer tissue samples (T1-T4) were positive for CacyBP/SIP expression, while the normal tissue samples (N1-N4) showed minimal expression. The colon cancer cell lines HT-29 and SW480 were positive for CacyBP/SIP expression, while the Lovo colon cancer cell line was negative. N, normal tissue; T, colon cancer tissue.

Our previous work showed that CacyBP/SIP is overexpressed in colon cancer tissue compared to normal tissues [12, 13]. To gain more evidence, we analyzed a larger array of cancerous tissue, adjacent benign tissue, and healthy control tissue by immunohistochemistry. CacyBP/SIP expression was detected in the cytoplasm and/or nucleus of 51% of colon cancer tissue (17 of 33), compared to only 19% of adjacent tissue (5 of 26; p < 0.05). In contrast, CacyBP/SIP staining was nearly undetectable in 10 normal colon tissue (Fig 1A and 1B). Consistnetly, western blot analysis showed CacyBP/SIP was overexpressed in cancerous colon tissue but undetectable in adjacent normal tissue (Fig 1C). CacyBP/SIP protein was also expressed in two of the three tested colon cancer cell lines HT29 and SW480 (Fig 1D). These results suggest that CacyBP/SIP overexpression may positively correlate with colon cancer.

### CacyBP/SIP promotes the growth of colon cancer cells under both basal and gastrin stimulated conditions

To study the role of CacyBP/SIP in proliferation of colon cancer cells, we examined the effect of CacyBP/SIP knockdown on the cell proliferation under either basal condition or treatment with gastrin, a hormone known to trigger colon cancer [19, 20].

**Fig 2:**
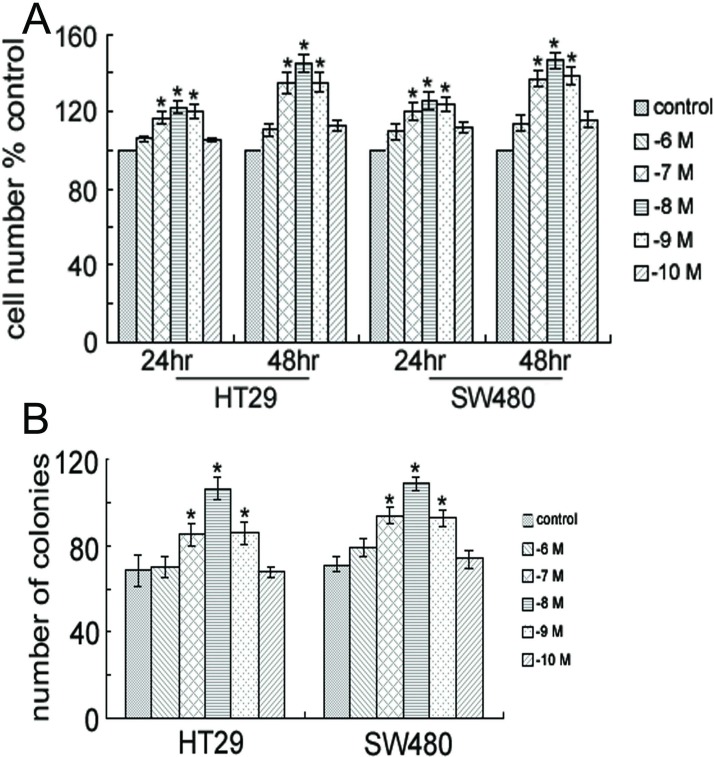
Gastrin promotes the growth of colon cancer cells. (A) Proliferation of HT29 and SW480 cells. Cells were treated with gastrin (10^−6^, 10^−7^, 10^−8^, 10^−9^, or 10^−10^ M) for 24 or 48 hours and their viability was determined by MTT assay. Columns, means; bars, F±SEM of three separate experiments in which each treatment was performed in five wells. *, P < 0.05 compared to control (without gastrin). (B) Gastrin-induced anchorage-dependent growth. Colony formation of HT29 and SW480 cells was assessed with or without the treatment of 10^−8^ M gastrin for 14 days; *, P < 0.05 compared to control (without gastrin).

Firstly, we determined the optimum concentration of gastrin which showed maximum stimulatory effect on the proliferation of colon cancer cells by MTT assay. Cells were grown in the medium containing gastrin at different concentrations 10^−6^, 10^−7^, 10^−8^, 10^−9^, and 10^−10^ mol/L. Similar to a previous report [19], proliferation of HT29 and SW480 cells was enhanced after 24 and 48 h of incubation with 10^−7^, 10^−8^, or 10^−9^ mol/L of gastrin (Fig 2A), and the greatest stimulatory effect was seen at 10^−8^ mol/L of gastrin. Thus, in the later experiments, we will use 10^−8^ mol/L gastrin as stimulant. Gastrin also dramatically enhanced anchorage-dependent growth, as indicated by colony formation in solid culture medium (Fig 2B).

The stimulatory effect of gastrin on the proliferation of colon cancer cells were further demonstrated by cell cycle analysis with flow cytometry. Cells were stained with PI after 48 h of treatment with 10^−8^ mol/L of gastrin, and then analyzed by flow cytometry. 70.93±0.55% and 62.17±0.75% of untreated HT29 and SW480 cells, respectively, were in the G1 phase, compared to 65.57±0.80% and 51.93±0.51% of gastrin-treated cells. This suggests that the G1 phase of treated cells was significantly shorter than that of the untreated cells in both HT29 and SW480 (*P* < 0.05; Table 1).

**Table 1:** Proportion of cells in the G0-G1 phases in the presence or absence of gastrin.

	HT29		HT29-CacyBPsi			SW480		SW480-CacyBPsi		
	G1	S	G1	S	*p* value	G1	S	G1	S	*p* value
Uninduced(%)	70.93±0.55	15.77±1.51	77.97±2.66	13.13±3.04	0.044	62.17±0.75	23.8±4.42	74.27±0.97	15.97±3.31	0.001
Gastrin-induced(%)	65.57±0.80	21.13±3.52	76.9±1.54	15.63±2.66	0.005	51.93±0.51	35.1±7.18	71.97±0.98	15.23±1.47	0.002
*p* value	0.009		0.62			0.005		0.075		

*P* value in the last row is calculated for same cell between different treatment, *P* value in the column is for the same treatment between CacyBP knockdown cell and control cell.

**Fig 3:**
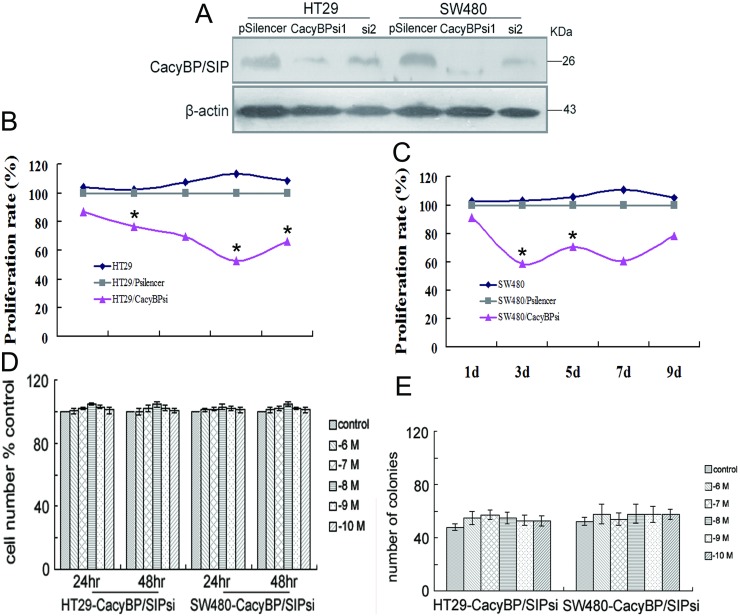
Knockdown of CacyBP/SIP expression slows cell growth in colon cancer cells. (A) Expression of CacyBP/SIP was reduced by CacyBP/SIPsi1 in both HT29- and SW480 cell lines. Control cell lines were established in HT29 and SW480 by stably transfected with a negative control siRNA transgene (pSilencer). Actin was used as an internal control. (B) Knockdown of CacyBP/SIP abolished the stimulatory effect of gastrin on the proliferation. This experiment was carried out as described in Fig 2A. (C) Inhibition of CacyBP/SIP expression results in decreased anchorage-dependent growth induced by gastrin. This experiment was carried out as described in Fig 2B. (D, E) Proliferation rate of stably transfected CacyBP/SIPsi cells are slower than the corresponding control cell lines for both HT29 and SW480. The relative proliferation rate was measured by MTT assay and normalized by HT29 or SW480 control cells for each day.

To examine whether CacyBP/SIP promotes colon cancer cell proliferation, we reduced the expression of CacyBP/SIP using short interfering (si) RNA. Two CacyBP/SIP-specific siRNA vectors, named CacyBP/SIPsi1 and CacyBP/SIPsi2, were constructed. After transfection and selection with G418 for 6 weeks, expression of CacyBP/SIP in the stably transfected cells was determined by Western blot. CacyBP/SIPsi1 largely reduced the the expression of CacyBP/SIP in both cell lines, while CacyBP/SIPsi2 had less substantial effect (Fig 3A). Consequently, cells stably transfected with CacyBP/SIPsi1 were chosen for subsequent experiments.

By cell cycle analysis with flow cytometry, under basal conditions, 77.97±2.66% of HT29-CacyBP/SIPsi1 cells compared with 70.93±0.55% of HT29 cells, and 74.27±0.97% of SW480-CacyBP/SIPsi1 cells compared with 62.17±0.75% of SW480 cells were in G0-G1 phase, respectively(*P* < 0.05; Table 1). This suggests that CacyBP/SIP promotes the cell cycle progression of these two colon cancer cells under the basal condition. Consistently, MTT assay showed that the cell proliferation is significantly decreased on the 3rd day when CacyBP/SIP was knockdown in both HT29 (Fig 3B) and SW480 (Fig 3C) cells.

More interestingly, knockdown of CacyBP/SIP abolished the stimulatory effect of gastrin on the proliferation of these two cancer cells. After 48 h of gastrin treatment, 76.9±1.54% of treated HT29-CacyBP/SIPsi1 cells was in the G1 phase compared with 77.97±2.66% of untreated cells. And 71.97±0.98% of treated SW480-CacyBP/SIPsi1 cells was in the G1 phase compared with 74.27±0.97% of untreated cells. The proportion of cells in G0-G1 was similar for untreated cells and gastrin-treated cells in both siRNA-expressing cell lines (*P* > 0.05; Table 1). Consistently, MTT assay showed that gastrin was unable to stimulate the proliferation of either HT29-CacyBP/SIPsi1 or SW480-CacyBP/SIPsi1 cells after 24 or 48 h (Fig 3D).

Colony formation of the CacyBP/SIP knockdown cells in solid culture medium was further studied. Under basal conditions, both HT29 and SW480 form fewer colonies after knockdown of CacyBP/SIP (Fig 3E). In addition, neither of the CacyBP/SIP knockdown cells showed increased anchorage-dependent growth following gastrin stimulation (Fig 3C). These results demonstrate that CacyBP/SIP promotes cell growth under both basal and gastrin stimulated condition.

### Gastrin stimulated nuclear translocation of CacyBP/SIP may be involved in the cell proliferation and cell cycle progression

**Fig 4:**
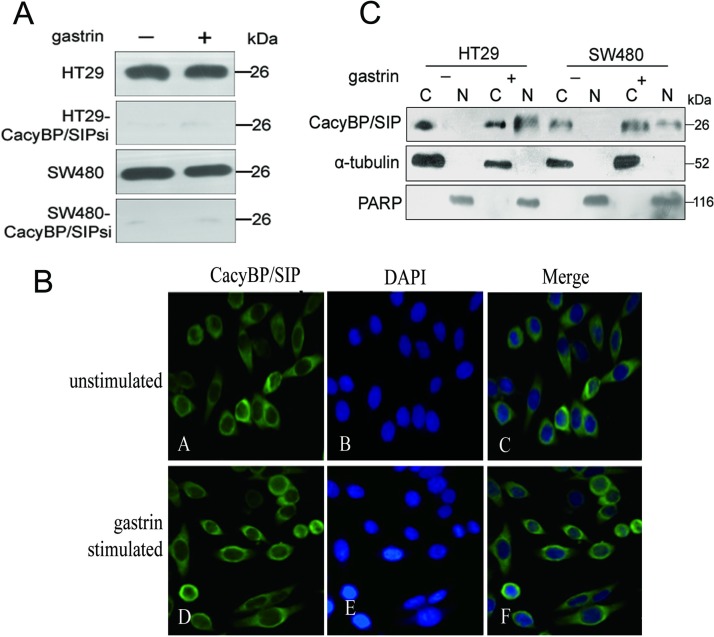
Gastrin induces CacyBP/SIP nuclear translocation and has no effect on its expression. (A) Gastrin has no apparent effect on CacyBP/SIP expression. The expression of CacyBP/SIP in HT29 and SW480 and the corresponding CacyBP/SIP knockdown cells were compared between basal and 10^−8^ mol/L gastrin stimulated for 8 h condition by western blot analysis. (B), CacyBP/SIP was translocated to the perinuclear region in SW480 cells. After stimulation (or not) with 10^−8^ mol/L gastrin for 8 h, SW480 cells were fixed and immunostained using CacyBP/SIP MAb (panels *a* and *d*); nuclei were labelled with PI (panels *b* and *e*). The merged images are shown in panels *c* and *f*. (C) The amount of CacyBP/SIP in nuclear extracts was examined before (-) and after (+) gastrin stimulation for 8 h. Cell lysates were prepared and probed with anti-CacyBP/SIP MAb, α-tubulin (cytosolic marker), and poly-ADP ribose polymerase (PARP, nuclear marker). C = cytoplasmic extract, N = nuclear extract.

One possible mechanism for CacyBP/SIP to mediate the stimulatory effect of gastrin on the cell proliferation is that gastrin can induce the expression of CacyBP. Fig 4A shows no obvious change in the expression of CacyBP/SIP in HT29, HT29-CacyBP/SIPsi1, SW480, or SW480-CacyBP/SIPsi1 cells following 10^−8^ mol/L gastrin treatment for 8 h.

Another interesting phenomenon is that CacyBP/SIP can translocate to the perinuclear region to be associated with the nucleus under several conditions [14–17]. The distribution of CacyBP/SIP was studied in gastrin stimulated or unstimulated SW480 cells by immunofluorescence using an anti-CacyBP/SIP MAb. In unstimulated cells, CacyBP/SIP was seen to be distributed throughout the cytoplasm (Fig 4Bc). This staining pattern was observed in the majority of cells examined. However, CacyBP/SIP were primarily distributed to the perinuclear region in gastrin stimulated cells (Fig 4Bf), suggesting that gastrin stimulation triggers the nuclear translocation of CacyBP/SIP. This is also supported by analysis of the relative abundance of nuclear and cytoplasmic CacyBP/SIP in colon cancer cells by Western blotting before and after 10^−8^ mol/L gastrin stimulation for 8 h. CacyBP/SIP was detected in the cytoplasm as well as the nucleus following gastrin stimulation, whereas it was found only in the cytoplasm in unstimulated cells (Fig 4C). It is possible that the nuclear translocation of CacyBP/SIP stimulated by gastrin itself may be playing an important role in colon cancer cells.

### CacyBP/SIP nuclear translocation promotes proteasome-mediated degradation of p27kip1 by interacting with Skp1

**Fig 5:**
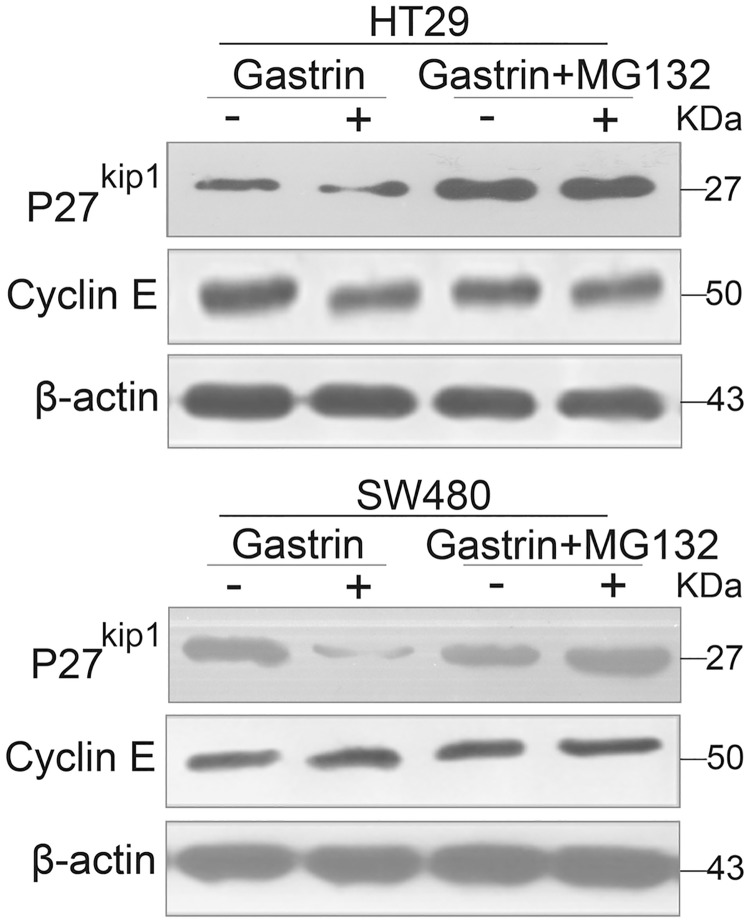
Gastrin induced proteasome-mediated degradation of p27^kip1^ in colon cancer cells. Western blot analysis of p27^kip1^ and Cyclin E in colon cancer cells after treatment with 10^−8^ mol/L gastrin in the absence or presence of MG132 (5 mM, 15 min pretreatment and co-treatment with gastrin).

**Fig 6:**
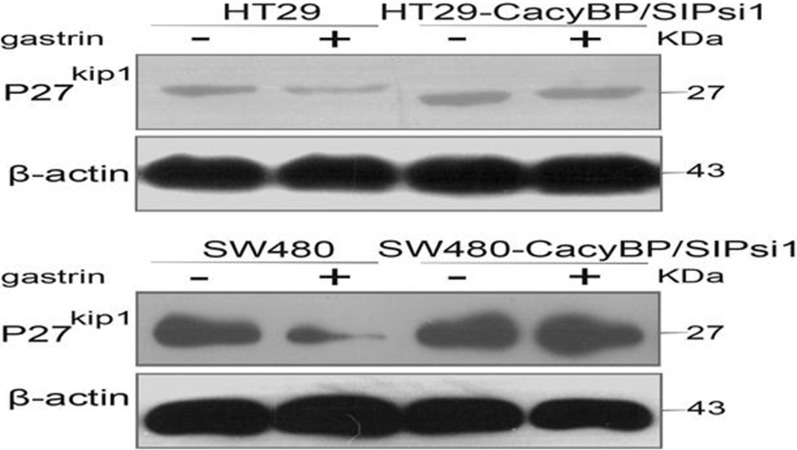
Gastrin could not induce the degradation of p27^kip1^ in CacyBP/SIPsi1 transfected cells. Western blot analysis of p27^kip1^ in colon cancer cells treated with or without gastrin. In HT29 and SW480 cells, the expression of p27^kip1^ obviously decreased after gastrin treatment. In HT29-CacyBP/SIPsi1 and SW480-CacyBP/SIPsi1 cells, the expression of p27^kip1^ showed no obvious change in response to gastrin.

CacyBP/SIP has been shown to be involved in ubiquitination mediated proteasome degradation pathway, through which p27^kip1^ is known to be degraded [21, 22]. Therefore we tested whether gastrin induced the degradation of p27^kip1^ in a proteasome-dependent manner using the 26S proteasome inhibitor MG132. As shown in Fig 5, p27^kip1^ levels decreased after exposure to gastrin. However, pre-treating cells with MG132, followed by co-incubation with MG132 and gastrin, blocked this decrease. This suggests that the 26S proteasome is indeed involved in the degradation of p27^kip1^. More importantly, gastrin could not induce the degradation of p27^kip1^ in HT29-CacyBP/SIPsi1 and SW480-CacyBP/SIPsi1 cells (Fig 6).

**Fig 7:**
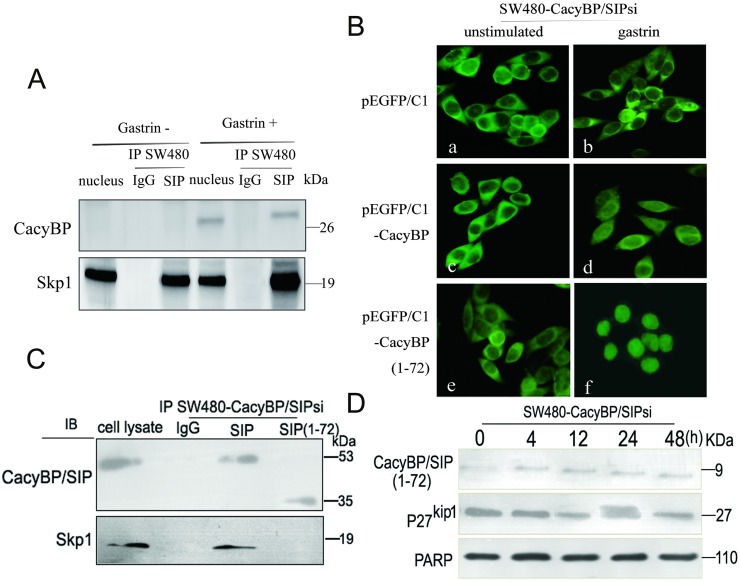
CacyBP/SIP interacts with the SCF component Skp1. (A) Physical interaction between Skp1 and CacyBP/SIP in colon cancer cells. Nuclear lysates from HT29 and SW480 cells were immunoprecipitated with monoclonal antibodies against CacyBP/SIP or control IgG. The immunoprecipitates were then probed with an anti-Skp1 antibody using ECL-based detection. (B) HT29 and SW480 cells were transiently transfected with plasmids pEGFP/C1 producing a GFP-tagged version of either full-length CacyBP/SIP or the truncation mutant CacyBP/SIP (1–72) lacking the C-terminal 155 amino acids. Cells transfected with empty vector pEGFP/C1, served as a negative control. Transfectants were treated or not with gastrin (10^−8^ mol/L, 4 h), and EGFP fluorescence was analyzed under a confocal laser microscope. *a*,*c* and *e* unstimulated cells; *b*,*d* and *f*, cells after gastrin stimulation for 8 h. (C) Transfected cell lysates were subjected to immunoprecipitation using either anti-CacyBP/SIP antibody or IgG as control. Immune complexes were analyzed by immunoblotting using anti-Skp1 antibody with ECL-based detection. (D) After treatment with 10^−8^ mol/L gastrin for 8 h, lysates from the transfected cells were evaluated by Western blot analysis. Equal amounts of total cellular protein (40 μg) were subjected to SDS-PAGE followed by Western blot analysis for p27^kip1^. PARP was used as a nuclear protein loading standard.

This result led us to ask whether CacyBP/SIP nuclear translocation enhances ubiquitin-mediated degradation of p27^kip1^ by regulating the ubiquitin pathway. Previous studies suggest that p27^kip1^ is the primary target of the SCF complex [23–26], and that CacyBP/SIP binds to SCF through the interaction between the C-terminal region of CacyBP/SIP and the adaptor protein Skp1 [4]. Thus, it is further tested whether gastrin increased the interaction between CacyBP/SIP and the SCF complex in gastrin stimulated cell nucleus. Nuclear lysates from cell treated with gastrin for 48 h were immunoprecipitated with anti-CacyBP/SIP, and the immunoprecipitated complexes were analyzed by anti-Skp1 immunoblotting (Fig 7A). Analysis of the CacyBP/SIP immunoprecipitates showed a band corresponding to the Skp1. Thus, we confirmed that CacyBP/SIP was binding to Skp1 under our experimental conditions.

To gather more direct evidence of whether interaction between CacyBP/SIP and Skp1 is involved in the gastrin-induced decrease in p27^kip1^ levels, we constructed a truncation mutant of CacyBP/SIP that was unable to interact with Skp1. This mutant possesses residues 1–72, and it lacks the C-terminal residues 73–229 previously shown to interact with Skp1. To minimize interference from endogenous CacyBP/SIP, the pEGFP/C1 plasmid encoding this mutant was transfected into SW480-CacyBP/SIPsi cells in which the expression of CacyBP/SIP was suppressed. In transiently transfected cells, this mutant was able to translocate to the nucleus after gastrin induction but failed to interact with Skp1 by co-immunoprecipitation. Furthermore, no change was detected in the levels of p27^kip1^ protein after gastrin induction (Fig 7B and 7D), suggesting an important role for interaction between CacyBP/SIP and Skp1 in the regulation of p27^kip1^.

## Discussion

Previously we showed that CacyBP/SIP is expressed at high levels in several cancers, including colon cancer. The present work not only confirmed this finding with a large set of samples, but also demonstrated an important role for CacyBP/SIP in promoting proliferation of human colon cancer. Knockdown of CacyBP/SIP retarded the growth of colon cancer cells under both basal and gastrin-treated conditions. Gastrin stimulates the nuclear translocation of CacyBP, leading to the proteasome-mediated degradation of p27^kip1^.

CacyBP/SIP is expressed at higher levels in cancerous colon tissue than in adjacent noncancerous tissues, and that its expression is undetectable in normal colon tissues. This supports the hypothesis that CacyBP/SIP expression levels may positively correlate with colon cancer. In HT29 and SW480 cell models, CacyBP/SIP knockdown decreased the basal cell proliferation rate and abolished stimulated cell proliferation by gastrin. Under normal conditions, gastrin treatment of human cancer cells significantly stimulated proliferation and colony formation, based on MTT and colony formation assays. However, this stimulation is not seen in cells in which CacyBP/SIP expression is strongly reduced by siRNA. Therefore, CacyBP/SIP overexpression is also functionally related to the colon cancer growth.

Although studies from our group and other groups have observed an interesting phenomena that CacyBP/SIP translocates to the nucleus in response to changes in intracellular Ca^2+^ concentration [9, 14]. The possible function of this translocation has never been studied in detail. In the present study, we find that CacyBP/SIP was translocated to the nucleus upon stimulation with gastrin, which is known to activate phospholipase C, resulting in the formation of inositol triphosphate and diacylglycerol, finally leading to the elevation of intracellular Ca^2+^ [27, 28]. Thus, gastrin may induce the nuclear translocation of CacyBP/SIP in a similar way as elevated intracellular Ca^2+^. Although the detailed mechanism remains to be explored, this study demonstrated that nuclear translocation of CacyBP/SIP has a correlation with cell growth by degradation of p27^kip1^ through enhanced interaction with SKP, a key component in the ubiquitination pathway.

The cell cycle is tightly regulated at G1-S and G2-M checkpoints by several protein kinases. The G1-S checkpoint is controlled by the CIP/KIP and INK4 family members of CDK inhibitors; including p21^waf1/cip1^, p27^kip1^, and p18^ink4c^, which bind to G1 Cyclin/CDK complexes and inhibit their catalytic activity, thus preventing the transition of cells from G1 to S phase [29, 30]. The CDK inhibitor p27^kip1^ was recently shown to play a critical role in the regulation of human cancer growth. For example, decreased expression of p27^kip1^ is frequently found in human colon cancer and is a strong indicator of poor prognosis [31]. Unlike other tumor suppressors such as p53 and RB, mutation or deletion of the p27^kip1^ gene is rarely observed in carcinogenesis in humans, suggesting that deregulation of p27^kip1^ expression in tumors is often due to post-transcriptional mechanisms [22]. Low p27^kip1^ levels in tumors are attributed to an increased rate of proteasome-mediated degradation [23, 32].

In the present study, we found that gastrin also works through proteasome-mediated degradation of p27^kip1^, since p27^kip1^ expression did not change after treating the cells with the proteasome inhibitor MG132. This is most likely mediated by the enhanced interaction between CacyBP/SIP and with Skp1, which is the adaptor protein of the SCF complex. When we truncated the part of CacyBP/SIP that binds Skp1, the resulting mutant did not bind Skp1 and abolished gastrin induced degradation of p27^kip1^. The expression of p27^kip1^ also remained unchanged under the treatment of gastrin when CacyBP/SIP was down-regulated by knockdown. Therefore we speculate that the 26S proteasome is indeed involved in the degradation of p27^kip1^.

It remains unclear for the moment how the interaction between CacyBP/SIP and Skp1 acts via the SCF-Skp2 ubiquitin E3 ligase complex to cause degradation of p27^kip1^. Since CacyBP/SIP has been proposed to act as a chaperone [4], it is tempting to speculate that the protein promotes the binding of Skp2 to the SKP1-CUL1-ROC1. Future studies should address this mechanistic question.

Taken together, the results in the present study suggest that nuclear translocaion of CacyBP/SIP has a correlation with growth of colon cancer cells, which in turn enhances ubiquitin-mediated degradation of p27^kip1^. Several important questions remain to be answered. Gastrin acts as a carcinogen by promoting cell proliferation, and it transmits proliferative signals from the cell membrane or cytoplasm to the nucleus [19, 20, 33]. However, the signaling pathway through which gastrin works is largely unknown. It is also unknown whether nuclear translocation of CacyBP/SIP transmits the growth signals of other factors or hormones?

Another question is whether the behavior and effects of CacyBP/SIP observed here are specific to colon cancer or whether they also apply to other types of cancer. [34,35],. We have detected CacyBP/SIP in many types of tumor tissues, and it is overexpressed in nasopharyngeal carcinoma, osteogenic sarcoma, and pancreatic cancer. However, previous data from our lab demonstrated that overexpression of CacyBP/SIP inhibits the growth of renal carcinoma cells and gastric cancer cells, suggesting that CacyBP/SIP has tumor-specific roles, similar to SIRT1, NF-κB, and TGF-beta [36–38].

In conclusion, these results suggest that CacyBP/SIP may be promoting growth of colon cancer cells by enhancing ubiquitin-mediated degradation of p27^kip1^. Our results provided new insights into the function of CacyBP/SIP nuclear translocation in regulating the cell cycle and the mechanism through which gastrin regulates cell proliferation. This work also provides a rationale for the use of inhibitors of the gastrin in patients with colon cancer.

## Supporting information

S1 Table Flow cytometry data analyses. (PDF)
