# Supplementary material for: CacyBP/SIP promotes the proliferation of colon cancer cells
Source: PLoS One. 2017 Feb 14;12(2):e0169959. doi: 10.1371/journal.pone.0169959 (PMC5308830; doi:10.1371/journal.pone.0169959)
Supplement: S1 Table — (PDF) [file pone.0169959.s001.pdf]

S1 Table：Flow cytometry data analyses

			1ST	2ND	3RD	ADVERGE	SD	*p* value	*p* value
HT29	uninduced	G1	70.3	71.3	71.2	70.93333333	0.550757055	0.009169793	0.044211127
		S	17.5	14.7	15.1	15.76666667	1.514375559		
	gastrin	G1	65.5	66.4	64.8	65.56666667	0.802080628		
		S	22.7	23.6	17.1	21.13333333	3.521836642		
HT29si	uninduced	G1	77.3	75.7	80.9	77.96666667	2.663331247	0.619854894	0.005465248
		S	16.4	12.6	10.4	13.13333333	3.035347317		
	gastrin	G1	75.2	78.2	77.3	76.9	1.539480432		
		S	16.3	17.9	12.7	15.63333333	2.663331247		
SW480	uninduced	G1	62.9	62.2	61.4	62.16666667	0.75055535	0.005002059	0.001113725
		S	18.7	26.3	26.4	23.8	4.417012565		
	gastrin	G1	51.5	51.8	52.5	51.93333333	0.513160144		
		S	42.6	34.4	28.3	35.1	7.17565328		
SW480si	uninduced	G1	74.5	75.1	73.2	74.26666667	0.971253486	0.074554701	0.001692808
		S	16.3	12.5	19.1	15.96666667	3.3126022		
	gastrin	G1	73.1	71.5	71.3	71.96666667	0.986576572		
		S	14.1	16.9	14.7	15.23333333	1.474222959		
